# Silicon Protection of Sugar Beet (*Beta vulgaris*) Against Field Dodder (*Cuscuta campestris*): Preliminary Analysis

**DOI:** 10.1002/pei3.70048

**Published:** 2025-03-30

**Authors:** Akbar Aliverdi, Hamed Mansouri

**Affiliations:** ^1^ Department of Plant Production and Genetics, Faculty of Agriculture Bu‐Ali Sina University Hamedan Iran; ^2^ Sugar Beet Research Department Hamedan Agricultural and Natural Resources Research and Education Center, AREEO Hamedan Iran

**Keywords:** biotic stress, haustorium, parasitic weed, silicon source

## Abstract

This preliminary study aimed to investigate the mitigation effect of silicon (Si) on field dodder‐induced stress in sugar beet. The experiment was conducted as a completely randomized design with three factors, including parasitic infection (non‐parasitized and dodder‐parasitized sugar beet), Si source (5 mM Si in the form of Na_2_SiO_3_ or K_2_SiO_3_), and Si application method (control, seed pretreatment, irrigation, and foliar spraying). Without Si, field dodder caused a 44.9% reduction in shoot biomass and a 57.5% reduction in root biomass. Although pretreating seeds with Si solutions accelerated emergence, it did not significantly influence any other traits measured in the sugar beet. Sugar beets that received Si through irrigation exhibited better protection against field dodder than those that were sprayed; furthermore, K_2_SiO_3_ proved to be more effective than Na_2_SiO_3_. Irrigating or spraying sugar beet with K_2_SiO_3_ reduced field dodder biomass by 60%–65%, while the reduction ranged from 20% to 35% with Na_2_SiO_3_. The highest lignin content was observed by watering and spraying dodder‐parasitized sugar beet with K_2_SiO_3_, resulting in a 4.2‐fold increase through watering and a 3.8‐fold increase through spraying. Field dodder infection led to increased activity of enzymes involved in scavenging reactive oxygen species, including catalase, guaiacol peroxidase, superoxide dismutase, and lipoxygenase in sugar beet. The application of Si further increased the activities of superoxide dismutase and lipoxygenase. This preliminary study suggests that irrigating with K_2_SiO_3_ can help reduce damage caused by field dodder in sugar beet. However, additional research is necessary to evaluate the crop's response at the field level.

## 1 Introduction

Approximately 4200 parasitic plant species have been identified and classified into 274 genera. While only 30 genera of these genera are known as parasitic weed species affecting agricultural plants, the most damaging species are classified into four key genera, including *Cuscuta*, *Arceuthobium*, *Orobanche*, and *Striga* (Mérillon and Ramawat 2020). Within the genus *Cuscuta*, which includes around 200 species, the field dodder ( *Cuscuta campestris* Yunck.) is particularly troublesome. This is due to its wide geographic distribution, broad host range, and ineffective control options (Dechassa and Regassa 2021). Field dodder can parasitize over 100 plant species, which include various crops or non‐parasitic weeds from different genera (Jones 2018). Notably, it lacks roots and leaves and has no photosynthetic ability. Instead, it develops root‐like structures called haustoria, which serve to absorb water, minerals, and other molecules from the primary vascular tissues of its host (Baráth et al. 2025). The presence of field dodder negatively impacts the growth and yield of its hosts. For instance, it can cause a 50% seed yield loss in soybean (Akhmurzaev et al. 2024), a 45% forage yield loss in alfalfa (Meighani et al. 2024), a 30% root yield loss in carrots (Konieczka et al. 2009), a 40% fruit yield loss in eggplant (Al‐Gburi et al. 2019), and a 40% yield loss in flax (Khaffagy et al. 2022).

Sugar beet ( *Beta vulgaris* L.), the second largest sugar source crop after sugarcane in the world, is another host for field dodder. Approximately 4.5 million ha were harvested worldwide in 2023 to obtain 281.1 million tons of sugar beet roots (FAO 2025). Scientific literature from different countries showed a 15%–41% root yield loss in sugar beet infected by field dodder, reducing sugar content by 1%–2.6% (Jafarzadeh et al. 2015; Hoseyni et al. 2018; Üstüner 2018).

Silicon (Si) is the second most abundant element on Earth after oxygen and is considered unnecessary for plant nutrition (Siuda et al. 2024). Nonetheless, it is present in plant tissues. Si‐enhanced sugar beet yield has been reported under no‐stress conditions (Siuda et al. 2024; de Lima et al. 2024). Several studies on sugar beet have demonstrated that Si can help mitigate both biotic and abiotic stresses, including those caused by insects (Amine et al. 2022; Yarahmadi et al. 2022), plant pathogens (Shabrawy and Rabboh 2020; Yassin 2015; Khan and Siddiqui 2020; Abdellatif et al. 2024), drought (Masri et al. 2024), salinity (Enan and Nemeat Alla 2024), heavy metals (Kabir et al. 2021), ammonium (Olivera‐Viciedo et al. 2024), and pesticides (Jain et al. 2021). Initially, the protective effects of Si were thought to be mechanical, resulting from its ability to form a physical barrier that strengthens the cell wall. However, further research has revealed that Si also provides biochemical protection to plants (Otolakoski et al. 2023; Pereira et al. 2024).

Research by Lukacova et al. (2019) has indicated that Na_2_SiO_3_ can provide Si‐based protection for tobacco ( *Nicotiana tabacum* ) against dodder ( *Cuscuta europaea* ). Similarly, Al‐Gburi et al. (2019) reported that Na_2_SiO_3_ offers protection for eggplant ( *Solanum melongena* ) against field dodder ( *C. campestris* ). However, no studies have explored this protection in other economically significant crops, such as sugar beet. Additionally, K_2_SiO_3_ has been shown to have lower salinity compared to Na_2_SiO_3_. In this study, we hypothesize that applying Si to the seeds, roots, or leaves of sugar beet can protect it against field dodder. Additionally, we propose that the choice of Si source (Na_2_SiO_3_ vs. K_2_SiO_3_) may influence this protective effect.

## 2 Materials and Methods

### 2.1 Experimental Layout

A greenhouse study was conducted using a three‐factor completely randomized design (2 × 2 × 4; *n* = 6), repeated twice in 2021 and 2022 at the Bu‐Ali Sina University in Iran. The first factor was parasitic infection, which included non‐parasitized and dodder‐parasitized sugar beet. The second factor was Si source, with two options: Na_2_SiO_3_ and K_2_SiO_3_, both applied at a concentration of 5 mM. The third factor was Si application method, which included four treatments: (1) Control: Seeds were soaked in distilled water (dH_2_O). (2) Seed pretreatment: Seeds were soaked in the Si solutions. (3) Irrigation: Seeds were soaked in dH_2_O, and the plants were watered with 100 mL of the 5 mM Si solution on first, second, third, and fourth weeks after emergence. (4) Foliar spraying: Seeds were soaked in dH_2_O, and the plants were sprayed with the 5 mM Si solutions to wet the entire leaf surface on first, second, third, and fourth weeks after emergence. A non‐ionic surfactant, alkyl aryl polyglycol ether (Citogate), was added to the Si spray solutions at a concentration of 0.1% v/v.

### 2.2 Plant Growth

The seeds of sugar beet (cv. Shokofa, SBSI034, a diploid monogerm variety resistant to rhizomania and cyst nematode released in 2015) were initially soaked in a 5% NaOCl solution for 1 min, dH_2_O‐washed, and soaked in the dH_2_O (for the Si application methods of control, root, foliar) or 5 mM Si solutions (for the Si application method of seed) for 24 h at room temperature. Then, they were taken out of the solutions and placed on a Whatman filter paper to dry superficially. Then, four seeds were planted in each 5‐kg pot at a depth of 1 cm. The soil was loamy, consisting of 25% clay, 46% silt, and 29% sand, with 0.82 dS/m EC, 7.6 pH, 0.9% OM, 13.6 ppm P, 240 ppm K, and 0.09% N. The pots were placed in the greenhouse with an average temperature of 22°C/14°C, relative humidity of 20%/35% day/night, and a 12‐h natural photoperiod. They were watered evenly every 4 days, and after the seedlings emerged, they were thinned to two plants per pot.

One‐year‐old dodder seeds were collected from dodder‐infected sugar beet fields in the region. To ensure germination, the seeds were treated by a seed dormancy‐breaking protocol (Zare and Porameri 2021) that involved soaking them in sulfuric acid (98%) for 10 min. Then, the seeds were washed with dH_2_O and placed on filter paper to dry superficially. One week after the sugar beet seedlings emerged, the seeds of field dodder were planted at a depth of 0.5 cm, with a density of 10 seeds per pot, corresponding to the dodder‐parasitized sugar beet. They were thinned to two plants per pot after emergence.

### 2.3 Measurements

#### 2.3.1 Plant Biomass

Ten weeks after the emergence of sugar beet, the stems of field dodder were harvested from sugar beet leaves. Subsequently, the shoots and roots of sugar beet were harvested. Plant materials were then dried in an oven (65°C, 48 h) and weighed.

#### 2.3.2 Shoot Si Content

Madany et al. (2020) were followed to measure the shoot Si content of sugar beet using the following procedure: 500 mg of dry leaves were added to 10 mL of 0.6 M HCl + 1.5 M HF. After 1 h, 40 mL dH_2_O were added and stirred for 20 min. One hundred milligrams of the supernatant were added to 2 mL of (NH_4_)_6_Mo_7_O_24_. After 5 min, 4 mL of 0.1 M HOC(CH_2_CO_2_H)_2_ was added, and with dH_2_O, the volume was made up to 10 mL. The absorbance of the sample was then measured at 400 nm using a UV–visible spectrophotometer (DS5 Dual Beam Model).

#### 2.3.3 Shoot Lignin Content and Lignin Biosynthesis Enzyme

Madany et al. (2020) was followed to determine the lignin content in sugar beet shoots using the following procedure: One hundred milligrams of dry leaves were ground, passed through a 25‐mesh screen, and added to 2 mL of ethanol (95%). After centrifugation (14,000×*g*, 3 min), the pellet was taken out and washed with 2 mL of C_2_H_5_OH and C_6_H_14_ (1:2, v/v), air‐dried, re‐washed with 2 mL of 1:3 (v/v) C_2_H_3_BrO:CH_3_COOH, heated in a Bain Marie oven (70°C, 30 min), and cooled at room temperature. After cooling, 0.1 mL of 7.5 M HONH_2_·HCl and 0.9 mL of 2 M NaOH were added, and with CH_3_COOH, the volume was made up to 10 mL. After centrifugation (1000×g, 5 min), the absorbance of the sample was measured at 280 nm.

Madany et al. (2020) were followed to determine the phenylalanine ammonia‐lyase (PAL) activity in sugar beet shoots using the following procedure: 300 mg of fresh leaves were nitrogen‐frozen and then mixed with 6.5 mL of 50 mM Tris–HCl buffer pH 8.8 + 15 mM HSCH_2_CH_2_OH + 1 mM ethylenediaminetetraacetic acid (EDTA). The mixture was homogenized and centrifuged (20,000×g, 20 min). The supernatant was passed through an Ultracel‐50 membrane. The absorbance of the sample was recorded at 290 nm.

#### 2.3.4 Reactive Oxygen Species Scavenging Enzymes

Hasanuzzaman et al. (2018) were followed to measure reactive oxygen species (ROS)‐scavenging enzymes in sugar beet shoots using the following procedure. To measure guaiacol peroxidase (POX) activity, 1 g of fresh leaves was added to 3 mL of 0.1 M KH_2_PO_4_ buffer pH 6.8 + 0.1 mM EDTA. After homogenizing the mixture, it was filtered through a layer of cheesecloth. The resulting homogenate was centrifuged (16,000×g, 15 min). Twenty‐five microliters of the supernatant were mixed with 2 mL of 50 mM KH_2_PO_4_ buffer pH 6.8 + 20 mM guaiacol + 20 mM H_2_O_2_ and heated in a Bain Marie oven (70°C, 30 min). After heating, 0.5 mL of sulfuric acid (5%) was added. The absorbance of the sample was recorded at 480 nm. To measure the catalase (CAT) activity, 1 g of fresh leaves was added to 3 mL of 0.1 M KH_2_PO_4_ buffer pH 6.8 + 0.1 mM EDTA. The mixture was homogenized and then filtered through a layer of cheesecloth. Subsequently, the homogenate was centrifuged (16,000×g, 15 min). Fifty microliters of the resulting supernatant were added to 3 mL of 50 mM KH_2_PO_4_ buffer pH 7.0 + 20 mM H_2_O_2_. The absorbance of the sample was recorded at 240 nm. To measure the superoxide dismutase (SOD) activity, 1 g of fresh leaves was added to 3 mL of 0.1 M KH_2_PO_4_ buffer pH 6.8 + 0.1 mM EDTA. The mixture was homogenized and then filtered through a layer of cheesecloth. Subsequently, the homogenate was centrifuged (16,000×g, 15 min). Fifty microliters of the supernatant were mixed with 3 mL of 50 mM KH_2_PO_4_ buffer pH 7.8 + 13 mM L‐methionine + 100 μM EDTA + 75 μM p‐nitroblue tetrazolium chloride + 2 μM riboflavin and subjected to a 30 W fluorescent lamp for 5 min. The absorbance of the sample was recorded at 560 nm. To measure the lipoxygenase (LOX) activity, 1 g of fresh leaves was mixed with 3 mL of 50 mM KH_2_PO_4_ buffer pH 7. After homogenizing the mixture, it was filtered using a Whatman filter paper. From the filtrate, 100 μL were taken and added to 3 mL of 100 mM linoleic acid + 0.1% v/v Tween‐20 + 200 mM KH_2_PO_4_ buffer pH 6.5. The absorbance of the sample was recorded at 234 nm.

#### 2.3.5 H_2_O_2_ and Malondialdehyde Content

Hasanuzzaman et al. (2018) were followed to determine the shoot H_2_O_2_ and malondialdehyde (MDA) content of sugar beet using the following procedure. To determine the H_2_O_2_ content, 500 mg of fresh leaves were added to 5 mL of 0.1% (w/v) trichloroacetic acid (TCA). After homogenizing the mixture, it was centrifuged (10,000×g, 15 min). Five hundred microliters of the supernatant were mixed with 0.6 mL of 100 mM KH_2_PO_4_ buffer pH 7.0 + 1 M potassium iodide. The absorbance of the sample was recorded at 390 nm. To determine the MDA content, 500 mg of fresh leaves were added to 5 mL of 0.1% (w/v) TCA. After homogenizing the mixture, it was centrifuged (10,000×g, 10 min). Subsequently, 1 mL of the supernatant was added to 4 mL of 20% TCA + 0.5% thiobarbituric acid. This mixture was heated in a Bain Marie oven (95°C, 30 min) and allowed to cool at room temperature. After centrifugation (10,000×g, 10 min), the absorbance of the sample was recorded at 532 nm.

### 2.4 Data Analysis

The Shapiro–Wilk test was performed to assess the normality of residuals. Since no interaction was observed, data were averaged over the 2 years and analyzed using a three‐way analysis of variance (ANOVA) with PROC GLM in the SAS (v9.4 SAS Institute Inc., Cary, NC, USA). The Tukey test separated the means at *⍺* = 5%. In instances where there was a main effect of a factor or a two‐ or three‐way interaction among the factors, the relevant means were compared and displayed. The principal component analysis (PCA) method was utilized to reduce the dataset's variables to a limited number of principal components (PCs), effectively summarizing the information. A biplot analysis was conducted based on the PCA in the R (v2.1.0, Foundation for Statistical Computing, Vienna, Austria) to illustrate the relationship between the selected PCs and various treatments.

## 3 Results

### 3.1 Plant Biomass

**FIGURE 1:**
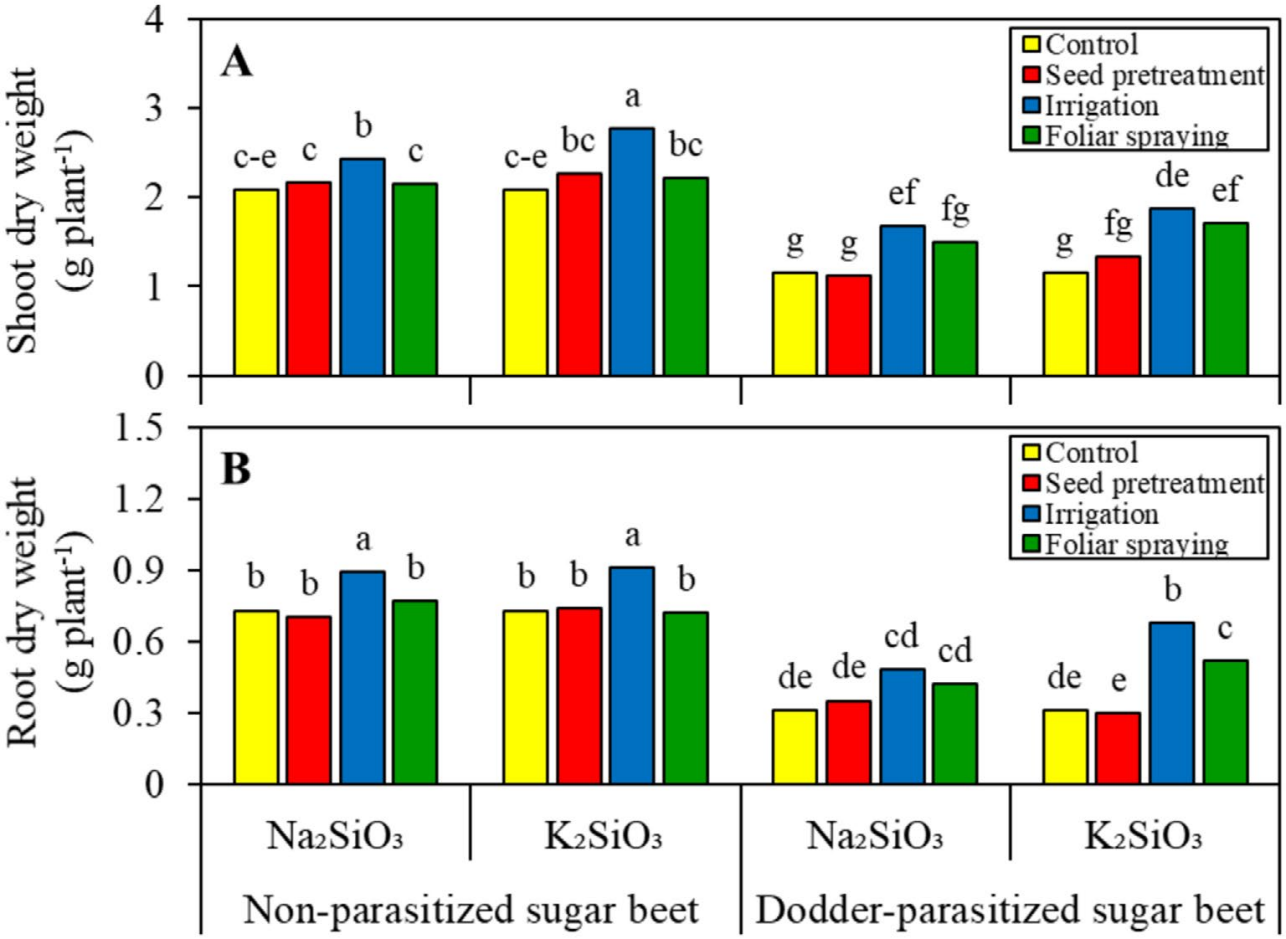
Effect of two silicon sources and four application methods on shoot (A) and root (B) dry weight of non‐parasitized and dodder‐parasitized sugar beets. Means followed by the same letter are not significantly different based on the Tukey test at *p* < 0.05.

Under conditions without Si, field dodder caused a reduction of 44.9% in shoot dry weight (Figure 1A) and 57.5% in root dry weight (Figure 1B). However, soaking seeds in the Si solutions did not have any impact on the growth of sugar beet, regardless of the level of parasitic infection. Unlike Na_2_SiO_3_, which had no effect, K_2_SiO_3_ foliar application to non‐parasitized sugar beet increased sugar beet shoot and root dry weight by 32.7% and 40.3%, respectively. Regardless of the level of parasitic infection, watering the plants with the Si solutions increased the shoot and root dry weight of sugar beet. Watering non‐parasitized sugar beets with Na_2_SiO_3_ and K_2_SiO_3_ had a similar effect on root growth of sugar beet (17.9% vs. 19.7%). However, K_2_SiO_3_ demonstrated a greater effect on shoot growth, with a 13.9% increase compared to a 24.5% increase for K_2_SiO_3_. Watering parasitized sugar beets with Na_2_SiO_3_ and K_2_SiO_3_ had a similar effect on shoot growth of sugar beet (31.5% vs. 38.5%). K_2_SiO_3_ was more effective for root growth, resulting in a 35.3% increase for Na2SiO3 compared to a 54.4% increase for K_2_SiO_3_. Overall, K_2_SiO_3_ promoted greater growth improvement than Na_2_SiO_3_. Dodder‐induced stress to sugar beet was mitigated by watering with K_2_SiO_3_.

**FIGURE 2:**
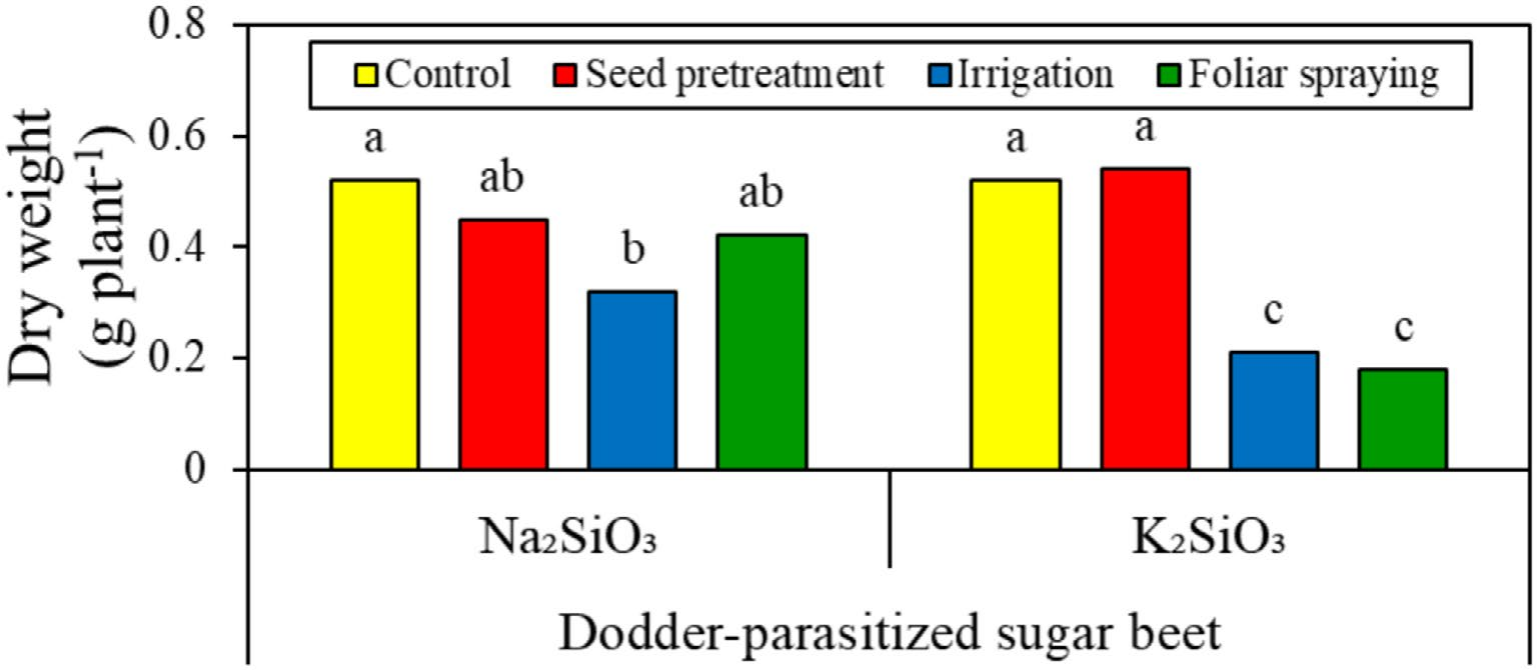
Effect of two silicon sources and four application methods on the biomass of field dodder parasitizing sugar beet. Means followed by the same letter are not significantly different based on the Tukey test at *p* < 0.05.

Soaking sugar beet seeds in the Si solutions—regardless of their source—did not have any effect on the biomass of field dodder (Figure 2). While other Si application methods did not produce significant differences among themselves, they generally reduced field dodder biomass. When Si was applied through root or foliar methods, K_2_SiO_3_ was more effective than Na_2_SiO_3_ in reducing the biomass of field dodder. Specifically, K_2_SiO_3_ applications reduced field dodder biomass by 60%–65%. The range for K_2_SiO_3_ applications was between 20% and 35%.

### 3.2 Shoot Si Content

**FIGURE 3:**
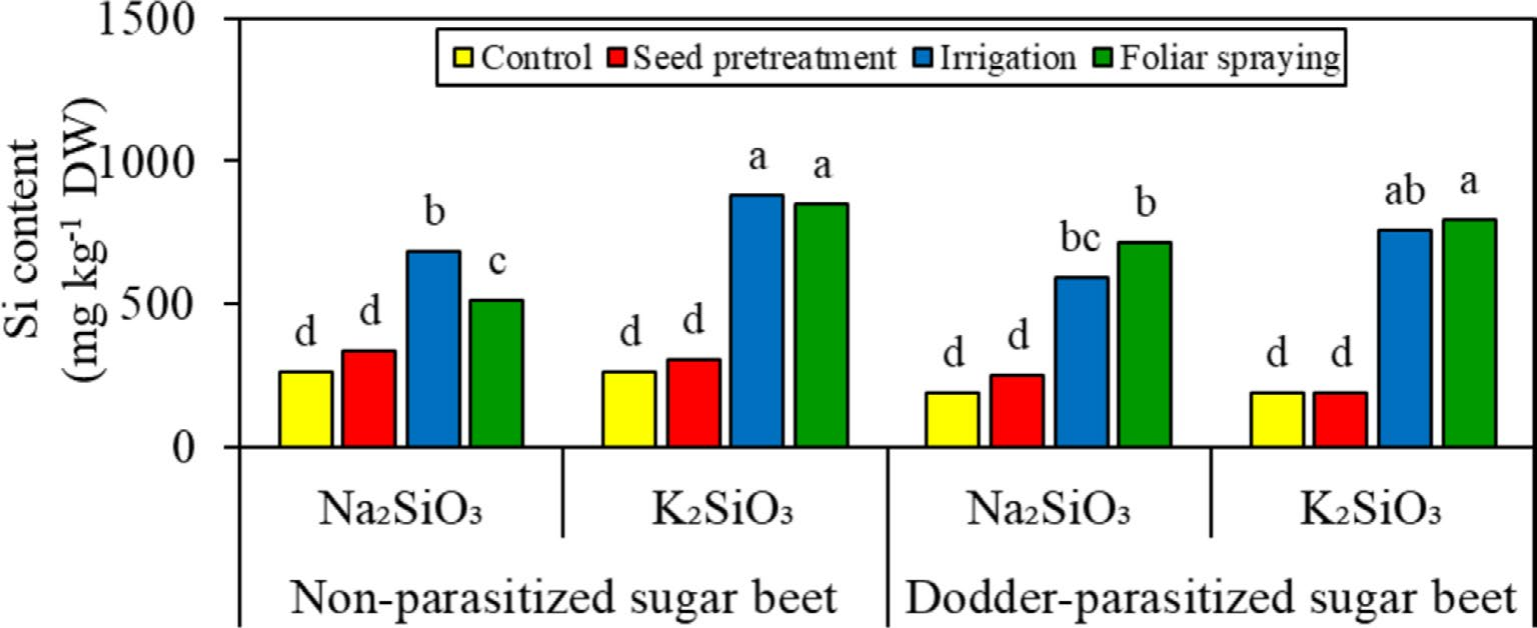
Effect of two Si sources and four application methods on the shoot Si content of non‐parasitized and dodder‐parasitized sugar beet. Means followed by the same letter are not significantly different based on the Tukey test at *p* < 0.05. DW, dry weight; Si, silicon.

Aside from foliar application of Na_2_SiO_3_, the Si content in the shoots of sugar beet was not affected by parasitic infection (Figure 3). In all levels of parasitic infection and Si source, soaking the seeds in Si solutions did not alter the shoot Si content of the sugar beets. However, watering or spraying the plants with the Si solutions increased the shoot Si content of sugar beet. Notably, watering the plants with Si solutions resulted in a higher shoot Si content (25.5%) in non‐parasitized sugar beet treated with Na_2_SiO_3_ than spraying the plants with Si solutions. Moreover, in each level of parasitic infection and Si application method, the plants treated with K_2_SiO_3_ exhibited a higher shoot Si content than those treated with Na_2_SiO_3_.

### 3.3 Shoot Lignin Content and Lignin Biosynthesis Enzyme

**FIGURE 4:**
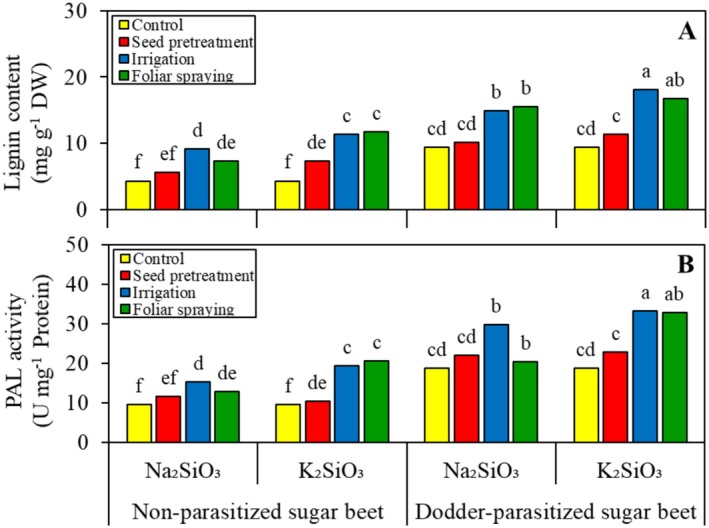
Effect of two silicon sources and four application methods on the shoot lignin content (A) and phenylalanine ammonia‐lyase (PAL) activity (B) of non‐parasitized and dodder‐parasitized sugar beets. Means followed by the same letter are not significantly different based on the Tukey test at *p* < 0.05. Each unit (U) of PAL activity equals the production of 1 μM C_6_H_5_‐CH=CH‐COOH min^−1^.

In the absence of Si, field dodder led to a 2.1‐fold increase in the lignification of sugar beet shoots (Figure 4A). Except for soaking seeds in K_2_SiO_3_ solution, soaking seeds in the Si solutions did not affect the lignification of sugar beet shoots, regardless of the level of parasitic infection and Si source. Other methods of applying Si to both non‐parasitized and dodder‐parasitized sugar beet plants resulted in increased lignification of sugar beet shoots. The effectiveness of these methods varied depending on the Si source, with K_2_SiO_3_ generally being the most effective. The highest lignin content was observed in dodder‐parasitized sugar beet that was irrigated and sprayed with K_2_SiO_3_, resulting in a 4.2‐ and 3.8‐fold increase in lignification compared with non‐parasitized sugar beet grown in Si‐free conditions.

The ANOVA results indicated that the PAL activity in sugar beet shoots was significantly influenced by all main factors: parasitic infection, Si source, and Si application method. Furthermore, the three‐way interaction among these factors was also significant. Under Si‐free conditions, field dodder caused a 1.9‐fold increase in the shoot PAL activity of sugar beet (Figure 4B). Unlike other Si application methods, soaking seeds in the Si solutions did not affect PAL activity in either non‐parasitized or dodder‐parasitized sugar beet. Additionally, the Si source did not affect PAL activity in non‐parasitized sugar beet. When dodder‐parasitized sugar beet was watered or sprayed with K_2_SiO_3_, the PAL activity was higher than that of those treated with Na_2_SiO_3_.

### 3.4 ROS Scavenging Enzymes

**FIGURE 5:**
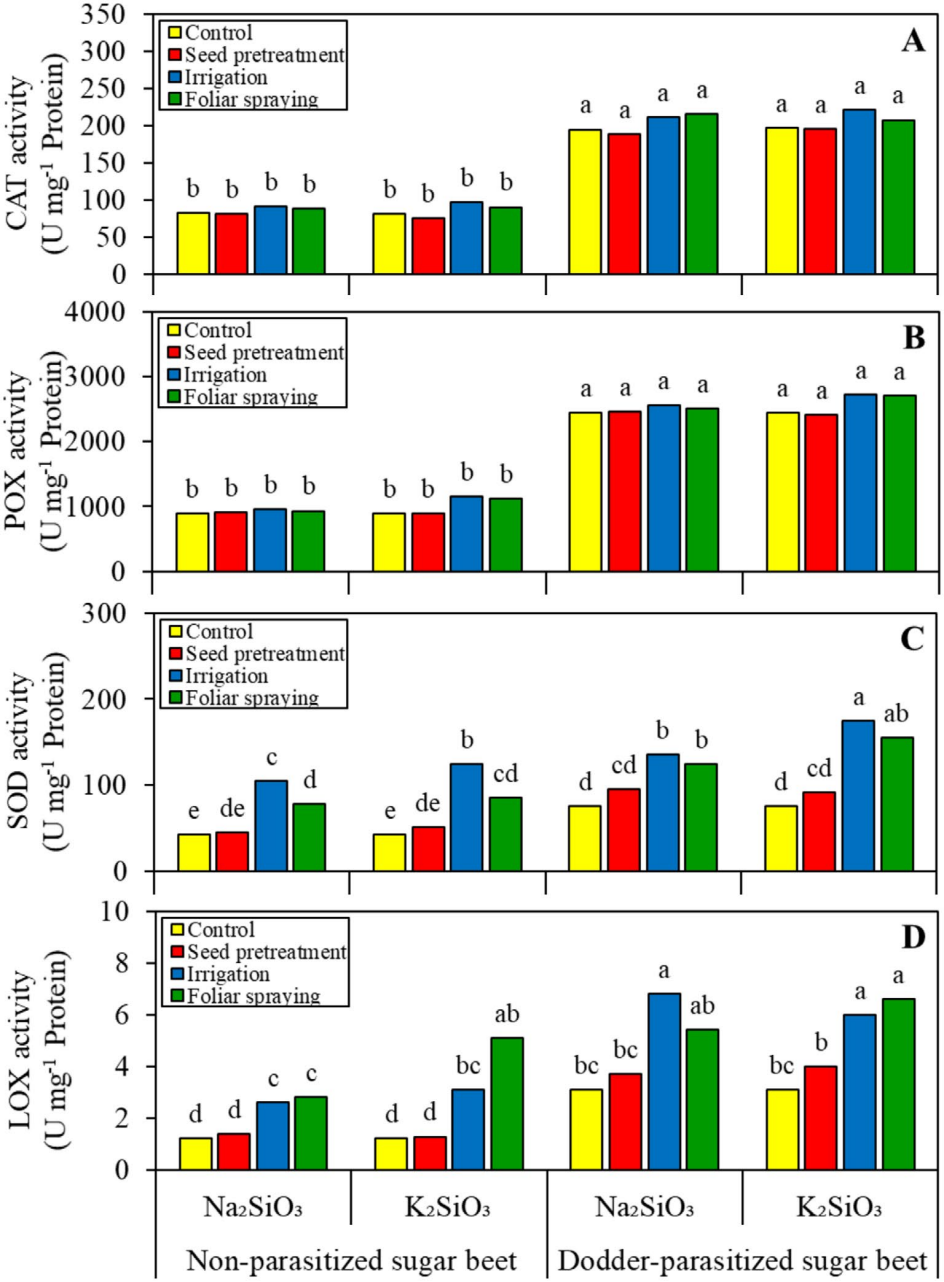
Effect of two silicon sources and four application methods on the shoot catalase (CAT) (A), guaiacol peroxidase (POX) (B), superoxide dismutase (SOD) (C), and lipoxygenase (LOX) (D) activity of non‐parasitized and dodder‐parasitized sugar beets. Each unit (U) of CAT activity equals the decomposition of 1 μM of H_2_O_2_ per minute^−1^. Each U of POX activity equals the change of one absorbance unit per mL^−1^ enzymatic extract. Each U of SOD activity equals the 50% enzymatic inhibition of nitroblue tetrazolium photoreduction. Each U of LOX activity equals 1 μM of hydroperoxide formed per minute^−1^. Means followed by the same letter are not significantly different based on the Tukey test at *p* < 0.05.

The ANOVA results indicated that the activity of CAT and POX was significantly affected only by the main effect of parasitic infection. Other factors and interactions did not show significant effects. In dodder‐parasitized sugar beet, the CAT (Figure 5A) and POX (Figure 5B) activities were found to be notably higher than those of non‐parasitized sugar beet, with increases of 2.3‐fold and 2.7‐fold, respectively.

In the analysis of SOD and LOX activity, the ANOVA revealed a significant three‐way interaction among parasitic infection, Si source, and Si application method. Soaking sugar beet seeds in the Si solutions, regardless of their source, did not impact SOD (Figure 5C) and LOX (Figure 5D) activities in the leaves. When sugar beet was not dodder‐parasitized, watering them with Si solutions resulted in higher SOD activity in the leaves than spraying them with Si solutions. However, there was no difference in SOD activity between these two methods when the sugar beet was dodder‐parasitized. In each level of parasitic infection, irrigating sugar beet plants with K_2_SiO_3_ resulted in higher SOD activity in the leaves than with Na_2_SiO_3_. This difference was not observed with foliar applications. However, Si irrigation and foliar applications did not differ in their ability to increase LOX activity in the leaves of sugar beet across all levels of parasitic infection and Si source.

### 3.5 H_2_O_2_ and MDA Content

**FIGURE 6:**
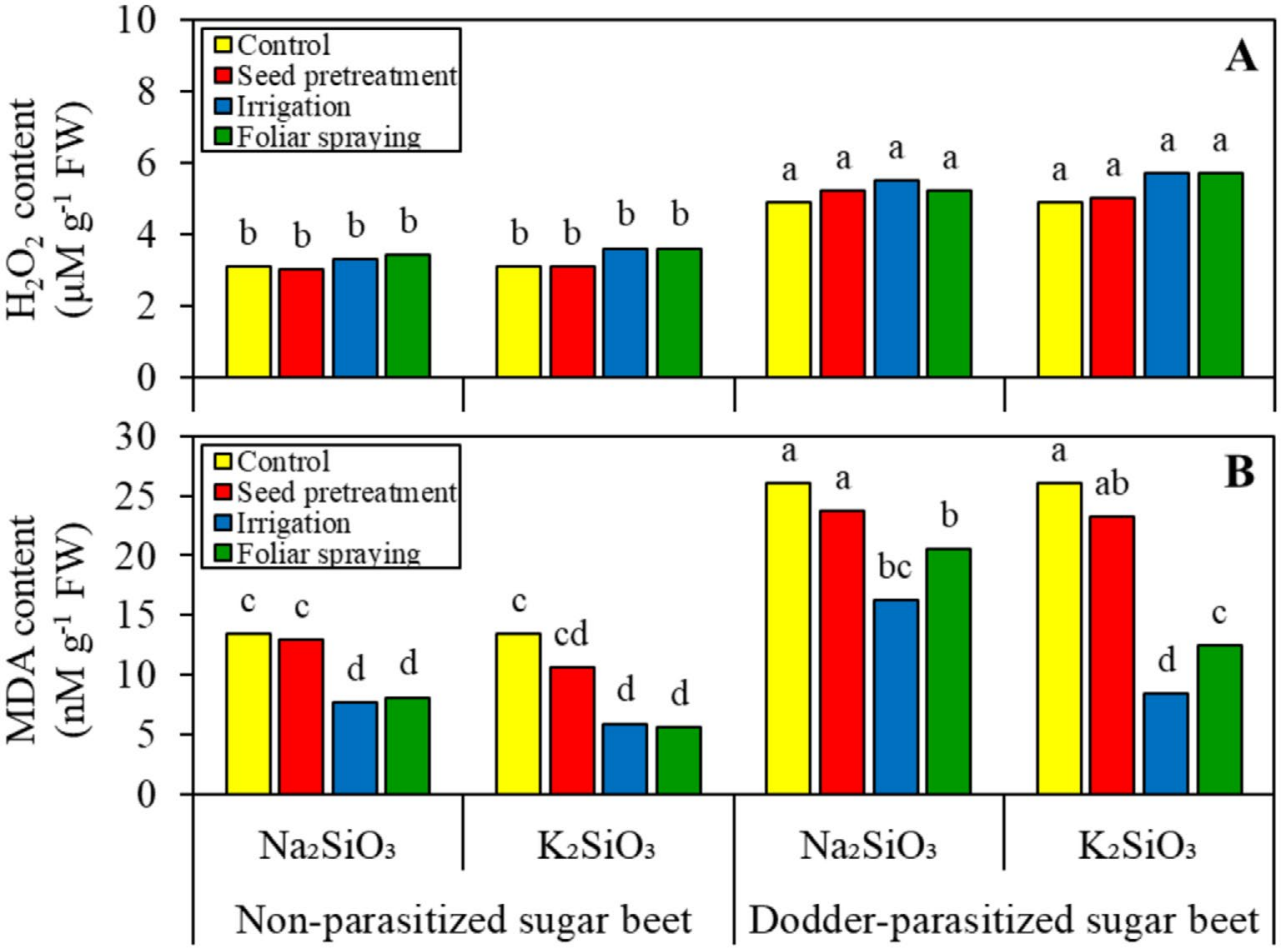
Effect of two silicon sources and four application methods on the shoot H_2_O_2_ (A) and malondialdehyde (MDA) (B) content of non‐parasitized and dodder‐parasitized sugar beets. Means followed by the same letter are not significantly different based on the Tukey test at *p* < 0.05.

The ANOVA results indicated that the H_2_O_2_ content was significantly influenced only by the main effect of parasitic infection. Other factors and interactions did not show significant effects. Dodder‐parasitized sugar beets contained 52.9% more H_2_O_2_ than non‐parasitized ones (see Figure 6A).

In the analysis of the MDA content, the ANOVA revealed a significant three‐way interaction among parasitic infection, Si source, and Si application method. Under conditions without Si, field dodder caused a 1.9‐fold increase in the shoot MDA content of sugar beet (Figure 6B). Soaking seeds in the Si solutions did not change the shoot MDA content of sugar beet at all levels of parasitic infection and Si source. However, alternative Si application methods were effective in reducing the shoot MDA content of sugar beet. Except for K_2_SiO_3_ applied to dodder‐parasitized sugar beet, no significant difference was observed between Si irrigation and foliar applications in decreasing the shoot MDA content of sugar beet. In dodder‐parasitized sugar beet, the reduction of MDA content induced by Si was influenced by the Si source, with K_2_SiO_3_ proving to be the most effective. Watering dodder‐parasitized sugar beets with K_2_SiO_3_ reduced the MDA content to levels comparable to those observed in non‐parasitized sugar beet.

## 4 Discussion

Si is considered an unnecessary element for plant nutrition, as it is absorbed in the form of Si(OH)_4_ and transported through the xylem. It is primarily deposited as SiO_2_ in cell walls, particularly in the epidermis (Saitoh and Suga 2022). In this study, soaking seeds in Si solutions did not have an effect on any of the traits measured. This lack of impact may be related to the way Si is deposited; specifically, the Si that accumulates in the seeds may not transfer to the shoots. This finding was in contrast with the observations of Lukacova et al. (2019), who reported that priming tobacco seeds with 2.5 and 5 mM Si solutions improved their tolerance to dodder. However, based on our observations, soaking seeds in Si solutions resulted in faster emergence of sugar beet seedlings, occurring one to 2 days earlier. This supports the findings of Ayed et al. (2022) and Tan et al. (2025) regarding Durum wheat ( *Triticum turgidum* L.) and rice ( *Oryza sativa* L.). If field dodder reacts similarly to Si regarding seed germination, it may lead to higher density and damage to the crop, highlighting the need for further study.

Research has shown that Si can protect sugar beet from various biotic stresses, including insect pests, plant pathogens, and nematodes. For instance, Shabrawy and Rabboh (2020) found that foliar applications of K_2_SiO_3_ significantly reduced the severity of sugar beet powdery mildew (*Erysiphe betae*), which in turn improved the yield of sugar beet roots and other related parameters. Yassin (2015) found that soaking sugar beet seeds in K_2_SiO_3_ and Na_2_SiO_3_ solutions effectively reduced seedling damping‐off caused by *Rhizoctonia solani* by inhibiting mycelial growth and germination of sclerotia. Khan and Siddiqui (2020) reported that priming sugar beet seeds and applying a foliar spray of SiO_2_ nanoparticles significantly mitigated the stress from root‐knot nematodes, specifically *Meloidogyne incognita*. Additionally, the role of Si in improving crops from parasitic weed species has received limited attention. Lukacova et al. (2019) found that applying Na_2_SiO_3_ through seeds, soil, or foliar methods prevented field dodder from infecting tobacco. Al‐Gburi et al. (2019) found that the foliar application of Na_2_SiO_3_ reduced field dodder infection on eggplant by up to 80%. Madany et al. (2020) showed that irrigating tomatoes ( *Lycopersicon esculentum* ) with SiO_2_ nanoparticles significantly reduced the severity of infection from branched broomrape ( *Orobanche ramosa* ).

**FIGURE 7:**
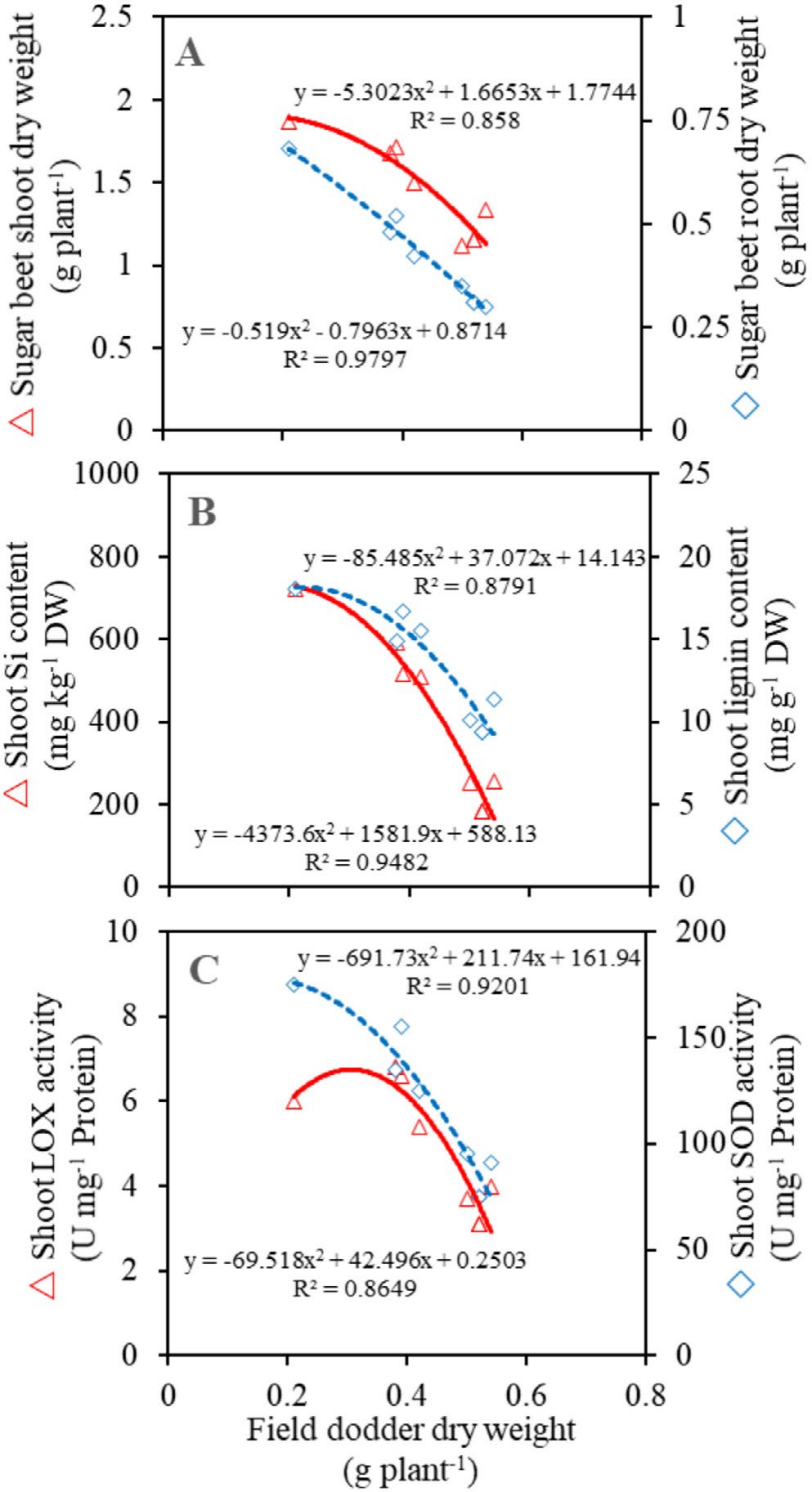
(A) A regression of shoot and root sugar beet dry weight over the dry weight of field dodder. (B) A regression of shoot Si and lignin content of sugar beet over the dry weight of field dodder. (C) A regression of shoot lipoxygenase (LOX) and superoxide dismutase (SOD) activity of sugar beet over the dry weight of field dodder. DW, dry weight; Si, silicon; U, unit.

This study demonstrated that watering or spraying sugar beet with Si enhances the sugar beet's resistance to field dodder. This improvement can be attributed to two mechanisms: physical and biochemical. Similar to previous studies (Lukacova et al. 2019; Al‐Gburi et al. 2019), our study showed the deposition of absorbed Si (Figure 3) in the epidermal cell walls, creating a mechanically stronger barrier against the penetration of field dodder's haustoria. Such an epidermal cell wall exhibits greater resistance to enzymatic degradation caused by the invasion of field dodder (Pereira et al. 2024). When Si is deposited beneath the cuticle, it forms a cuticle –Si double layer, further preventing the penetration of field dodder's haustoria and decreasing parasitic infection. Formation of cuticle –Si double‐layer has been reported to increase the thickness of tobacco leaves (Lukacova et al. 2019). As a result, a negative correlation was observed between the Si content in sugar beet shoots and the biomass of field dodder (Figure 7A).

**FIGURE 8:**
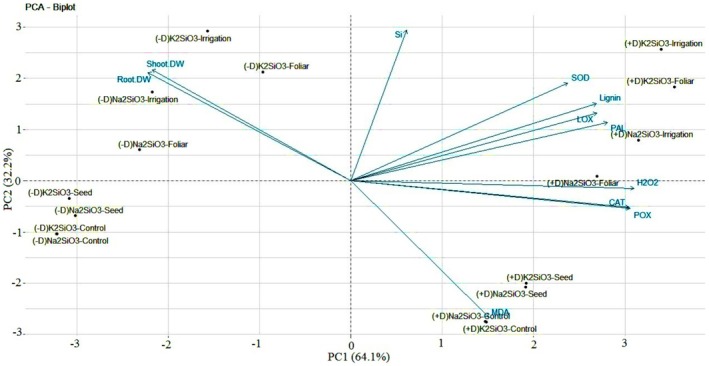
A biplot diagram of the first two principal components (PC1 and PC2) for traits measured in sugar beet in this study. Treatments included non‐parasitized (–D) and dodder‐parasitized (+D) sugar beets, both of which were treated with 5 mM solutions of Na_2_SiO_3_ and K_2_SiO_3_. These solutions were applied using four methods: control, seed pretreatment, irrigation, and foliar spraying.

It is suggested that biochemical resistance to field dodder, as regulated by Si, is more complex than merely physical resistance; this aspect has been strongly debated in recent years. Higher PAL activity, induced by Si (Figure 4B), is linked to increased lignin production (see Figure 4A,B). PAL converts L‐phenylalanine into trans‐cinnamic acid, which serves as a precursor for lignin and flavonoids. The accumulation of lignin in the epidermal cell walls may provide protective effects similar to those of Si in preventing the penetration of field dodder's haustoria (Pereira et al. 2024). Consequently, a negative correlation was observed between sugar beet shoot lignin content and field dodder biomass (Figure 7B). In this study, field dodder invasion induced oxidative stress in sugar beet by generating ROS, such as H_2_O_2_ (Figure 6A). These ROS can directly damage membrane lipids and increase MDA content (Figure 6B), which is recognized as an indicator of oxidative damage. This oxidative stress can trigger responses that lead to heightened activities of defense‐related enzymes, such as CAT, POX, SOD, and LOX, thereby helping the plants fend off further attacks from field dodder. The findings indicated that, although all ROS‐scavenging enzymes were more active in dodder‐parasitized sugar beet plants compared to non‐parasitized plants, the activities of SOD and LOX were particularly enhanced by Si (see Figure 5C,D). Furthermore, the effectiveness of these enzymes depended on the Si source used. A negative correlation was also found between the activities of LOX and SOD in sugar beet and the biomass of field dodder (Figure 7C), suggesting that the ability to scavenge ROS and mitigate their damaging effects is linked to the biochemical tolerance of sugar beet to field dodder. Similarly, increased activity of ROS‐scavenging enzymes has been reported in sweet basil ( *Ocimum basilicum* L.) when affected by field dodder (Ahmadi Mousavi et al. 2017). The PCA revealed that two PCs with eigenvalues greater than one were identified, explaining a total of 96.3% of the variance in the data. The coefficients of these PCs for various traits highlight the influence each trait has on the components. The first principal component (PC1) has an eigenvalue of 7.051, accounting for 64.1% of the total variance. It showed high positive coefficients for CAT, H_2_O_2_, lignin, LOX, PAL, POX, and SOD, while having high negative coefficients for root and shoot dry weight (Table 1). The second principal component (PC2) accounted for 25.59% of the total variance and had an eigenvalue of 3.542. In PC2, the coefficients for MDA were negative, whereas those for Si were positive. Based on the biplot analysis (Figure 8), the treatment group involving Dodder‐parasitized sugar beets, which received irrigation and foliar applications of both Na_2_SiO_3_ and K_2_SiO_3_, was positioned in the top right quadrant of the biplot space (indicating positive intervals for both PC1 and PC2). This group was characterized by high activities of CAT, LOX, PAL, POX, and SOD, as well as elevated contents of H_2_O_2_ and lignin. In contrast, the treatments involving non‐parasitized sugar beets, which received seed applications of both Na_2_SiO_3_ and K_2_SiO_3_, along with the control group, were located in the lower left quadrant of the biplot (indicating negative intervals for both PC1 and PC2). This group was associated with low enzyme activity and diminished contents of H_2_O_2_ and lignin.

**TABLE 1:** Principal component analysis (PCA) for traits measured in sugar beet in this study.

Traits	Principal components (PC)	
	PC1	PC2
Root dry weight	−0.712	0.679
Soot dry weight	−0.698	0.696
Si content	0.195	0.943
Lignin content	0.862	0.483
PAL activity	0.901	0.365
CAT activity	0.977	−0.167
POX activity	0.980	−0.173
SOD activity	0.761	0.611
LOX activity	0.864	0.424
H_2_O_2_ content	0.994	−0.047
MDA content	0.483	−0.854
Eigenvalue	7.051	3.542
Relative variance (%)	64.1	32.2
Cumulative variance (%)	64.1	96.3

Abbreviations: CAT, catalase; LOX, lipoxygenase; MDA, malondialdehyde; PAL, phenylalanine ammonia‐lyase; POX, guaiacol peroxidase; Si, silicon; SOD, superoxide dismutase.

A well‐balanced K^+^/Na^+^ ratio is essential for properly regulating various physiological processes in plants, such as stomatal function, enzyme activation, protein synthesis, cellular osmoregulation, oxidant metabolism, photosynthesis, and overall plant growth. To enhance stress tolerance, it is important to increase K^+^ uptake and reduce Na^+^ accumulation (Assaha et al. 2017). The current study revealed that sugar beets irrigated with K_2_SiO_3_ showed better protection against field dodder than those irrigated with Na_2_SiO_3_. This improvement is likely due to a favorable change in the K^+^/Na^+^ ratio. Similarly, a study conducted by Amine et al. (2022) found that applying Si in the soil led to a greater reduction in the larval density of the Egyptian cotton leafworm (*Spodoptera littoralis*) in sugar beet fields than foliar applications of Si. They also determined that K_2_SiO_3_ was more effective than Na_2_SiO_3_ and Mg_2_SiO_3_. Furthermore, Yarahmadi et al. (2022) found that foliar application of Ca_2_SiO_3_ reduced the larval density of the beet armyworm ( *Spodoptera exigua* ) in sugar beet fields, while foliar application of Na_2_SiO_3_ had no significant effect.

Some studies have demonstrated Si can induce the regulation of systemic signals—such as salicylic acid, jasmonic acid, and ethylene—which strengthen crops against biotic stresses, including parasitic weeds (Ghareeb et al. 2011; Ye et al. 2013). On the other hand, some studies have shown exogenous Si protects plants more effectively against abiotic stresses, including herbicides, by regulating these systemic signals that facilitate faster herbicide metabolism in plants (Kabir et al. 2021; Saudy and Mubarak 2015; Li et al. 2022; Soares et al. 2021; Tripthi et al. 2020). Although this study indicates that Si enhances sugar beet's resistance to field dodder, it is important to note that it may also diminish the effectiveness of herbicides against non‐parasitic weeds. This inconsistency warrants further investigation.

## 5 Conclusions

Our study demonstrated that soaking seeds in a 5 mM solution of Si did not affect any traits we measured in the sugar beet. Generally, sugar beets that were irrigated with Si exhibited better protection against field dodder compared to those that were sprayed with Si. The effectiveness of Si depended on the source used; K_2_SiO_3_ was more effective than Na_2_SiO_3_. The invasion of field dodder increased all ROS‐scavenging enzymes in sugar beet. Unlike CAT and POX, the activities of SOD and LOX were stimulated by Si. On average, the sugar beets irrigated with Si contained double the amount of lignin. These findings suggest that sugar beets develop both physical and biochemical mechanisms to enhance resistance through Si.

## Conflicts of Interest

The authors declare no conflicts of interest.

## Data Availability

The authors have nothing to report.
